# Interrelationships among College Students’ Perceptions of Smart Classroom Environments, Perceived Usefulness of Mobile Technology, Achievement Emotions, and Cognitive Engagement

**DOI:** 10.3390/bs14070565

**Published:** 2024-07-04

**Authors:** Yunpeng Ma, Mingzhang Zuo, Ruiyang Gao, Yujie Yan, Heng Luo

**Affiliations:** 1 Department of Education Science, Zhoukou Normal University, Zhoukou 466001, China; 2 Faculty of Artificial Intelligence in Education, Central China Normal University, Wuhan 430074, China

**Keywords:** smart classroom, mobile technology, achievement emotions, pride, anxiety, cognitive engagement

## Abstract

Cognitive engagement is a crucial factor that shapes successful learning outcomes, but our understanding of the factors that influence such engagement in the smart classroom context remains limited. This study aims to narrow this research gap by exploring the relationships among college students’ perceptions of the smart learning environment, perceived usefulness of mobile technology, achievement emotions, and cognitive engagement. A total of 1293 college students completed an online questionnaire survey, and 1076 valid responses were received. Structural equation modeling was used to analyze the interrelationships among these factors. The results revealed that students’ perceptions of the smart classroom environment and perceived usefulness of mobile technology as well as two achievement emotions (pride and anxiety) significantly impact cognitive engagement. Both pride and anxiety act as mediators in the relationships among perceptions of smart classroom environments, the perceived usefulness of mobile technology, and cognitive engagement, in which context the mediating effect of pride is stronger than that of anxiety. These findings have practical implications for instructors, who should focus on implementing strategies that promote positive achievement emotions when students use mobile technology in smart classrooms. Additionally, these findings can inform the design and construction of smart classroom environments. Moreover, our study has limitations due to reliance on online data collection and self-reported data, which may introduce biases and measurement errors. Future research should incorporate multimodal data and advanced technologies for a comprehensive assessment to better understand students’ engagement in smart learning environments, while also considering individual factors and the educational context to enhance the effectiveness of mobile technology in supporting students’ emotions and achievement.

## 1. Introduction

The integration of information and communication technologies (ICTs) into instruction is viewed as a way of promoting innovation, flexibility, and creativity [1], thereby providing students with dynamic and efficient learning ecosystems [2] and ensuring that they are actively engaged; this approach can lead to better learning experiences and outcomes [3]. Therefore, over the past few years, education systems worldwide have increased their investments in the process of integrating ICTs, leading to the rapid use and popularization of ICT-related tools [4] as well as significant changes in the learning environment [5]. The use of technology-based learning environments to facilitate teaching and learning has become a popular educational issue [6]. The rise of smart classroom environments serves as a significant indicator of colleges and universities’ attempts to adapt to emerging technologies such as the Internet of Things and Big Data Analytics [7].

A smart classroom environment refers to a physical classroom that employs advanced forms of educational technology that range beyond those available in traditional multimedia classrooms [8]; this concept has revolutionized classroom environments, transforming them into interactive and dynamic learning environments that feature modern technologies such as interactive whiteboards, tablets, and educational software [9,10]. The literature on this topic has suggested that smart classroom environments have the potential to disseminate knowledge effectively, promote innovation with regard to learning paradigms and teaching methods, facilitate the acquisition of educational resources, enhance teaching interactions, and collect feedback data [11,12,13]. In light of the many advantages associated with a smart classroom environment, the implementation of smart classrooms in government programs in various countries with the goal of enabling students to benefit from the advantages of the digital era [14] has become a prevalent trend in the evolution of higher education institutions [15]. This trend has led to increased demand for research on the positive impacts of smart classroom environments on student learning [16], primarily with regard to how students engage and learn in such a technology-supported environment [17].

Student engagement has been widely acknowledged as a crucial factor in attempts to facilitate learning in diverse environmental settings [7]. Engagement is a broad and complex phenomenon, has many definitions [18], and is regarded as a complex multidimensional structure [19,20]. For instance, Schaufeli et al. [21] relied on a student engagement questionnaire derived from the Utrecht Work Engagement Scale, which included three distinct subscales: “vigor”, “dedication”, and “absorption”. Drawing from flow theory, Shernoff et al. [22] conceptualized student engagement as a composite of “enjoyment”, “concentration”, and “interest”. Fredricks et al. [23] constructed a framework for engagement that included “behavioral”, “emotional”, and “cognitive” engagement; “social” engagement has also been included as a dimension in subsequent studies based on this framework [24]. Although previous research has been inconsistent with regard to the specific number and type of dimensions associated with student engagement, the view that cognitive, emotional, and behavioral engagement are three primary dimensions or subtypes of student engagement has become widespread [25,26].

Cognitive engagement is an important component of student engagement and refers to deliberate and willing effort on the part of learners to comprehend complex ideas and master difficult skills [23]. This dimension has been linked to learning most frequently [27,28] and has been viewed as a reliable indicator of learning and a first step toward the achievement of successful learning outcomes [29]. Higher cognitive engagement means that students are more likely to exhibit better academic performance [30]. Helping students engage in learning in different environments is an important issue. Previous studies have shown that student cognitive engagement in online, blended, or face-to-face learning environments is often influenced by various factors, such as the use of technology, preferences regarding the learning environment, and students’ own emotions [31,32,33,34,35,36,37,38,39]. As the prevalence and significance of smart classroom environments are increasing on a daily basis [40], understanding these factors and the interactions among them is crucial for educators who seek to create engaging and effective smart classrooms. However, little is known regarding student cognitive engagement and its influencing factors in the smart classroom context, which represents one gap in the literature on cognitive engagement. Therefore, the focus of this paper is to investigate how these influencing factors impact student cognitive engagement in smart classroom environments.

## 2. Literature Review and Hypothesis Development

### 2.1. Perceptions of the Smart Classroom Environment and Cognitive Engagement

The learning environment is an important factor influencing student engagement [22]. Students’ perceptions of the learning environment are often referred to as their learning environment preferences [16], which affect their engagement and contribute to their learning performance [41]. Sokmen’s research on the relationship between the learning environment and student engagement demonstrated that students’ perceptions of the learning environment significantly predict their cognitive engagement, and this predictive effect is stronger than those of student behavioral, emotional, and agentic engagement [42]. Previous research has indicated that student engagement is positively affected by technology-enhanced learning environments [18]. As a novel type of technology-enhanced learning environment, smart classrooms have become popular. Researchers have shown that such classrooms can enhance the cognitive engagement of students in the learning process [43]. For instance, Lu et al. [44] investigated the relationship between the preferences of college students regarding the smart classroom learning environment and their engagement, and the results revealed that smart classroom preferences such as those pertaining to inquiry learning, reflective thinking, and multiple sources could predict students’ cognitive engagement.

In conclusion, we propose the following hypothesis:

**H1.**  *Perceptions of the smart classroom environment (PE) have a positive effect on cognitive engagement (CE).*

### 2.2. Perceived Usefulness of Mobile Technology and Cognitive Engagement

The use of smartphones, tablets, and other wireless technology devices is becoming increasingly widespread among the general public; thus, social media has become an e-learning tool that is used by many teachers and students [45]. Moreover, smart classrooms typically feature multiple interactive technologies that are compatible with mobile devices [46]. Cebrián et al. [47] emphasized the importance of useful communication between digital and mobile devices with regard to learning quality in a smart classroom. In the smart classroom learning environment, the perceived usefulness of mobile technology is considered as the benefit of using mobile technology in learning and teaching [48]. Hence, schools and educational institutions are beginning to integrate mobile technologies seamlessly into smart classroom environments [10]. Mobile technologies have led to the emergence of unprecedented changes and opportunities for learning [43] and had positive effects on student engagement [33]. Previous studies have indicated that the use of mobile applications can target individual, task, and environmental factors that influence college students’ cognitive engagement [43,49]. Similarly, Alshuaibi et al. [50] reported that social media can enhance student cognitive engagement. In contrast, students may experience apathy and disengagement if they believe that mobile technology learning activities are meaningless [51].

The results of previous research have shown that perceived usefulness has a strong direct influence on student cognitive engagement. Thus, we propose the following hypothesis:

**H2.**  *The perceived usefulness of mobile technology (TU) has a positive effect on cognitive engagement.*

### 2.3. Achievement Emotions and Cognitive Engagement

Emotion represents a psychological response mechanism among humans, and it can significantly influence and regulate cognitive processes such as attention, perception, representation, memory, and thinking [52]. Emotions are critical to learning [53,54], and research on emotions in education settings has focused mostly on achievement emotions [55], which refer to multidimensional mental cycles that are objectively oriented and assessment-driven; achievement emotions are also tied directly to achievement activities or achievement outcomes [56]. Pekrun and Stephens [57] divided achievement emotions into two categories, i.e., positive emotions (such as enjoyment, pride, and hope) and negative emotions (such as anger, anxiety, shame, hopelessness, and boredom), based on their valence. Extensive empirical evidence has indicated that students experience a diverse array of discrete positive and negative achievement emotions in different classroom settings [58]. The positive psychology literature has suggested that positive emotions motivate students to participate actively in learning, while negative emotions prevent them from engaging in learning [59]; thus, positive emotions positively affect students’ engagement, while negative emotions are negatively associated with engagement [60]. Four distinct achievement emotions—enjoyment, pride, boredom, and anxiety—are highly salient in the academic context [61]. The current study focused on two discrete achievement emotions, i.e., pride and anxiety.

Pride is a constructive emotion that refers to a sense of satisfaction or pleasure that emerges from personal accomplishments [62] and is particularly significant and strong in academic situations [60]. Pride is often viewed as a reflection of the individual’s strong sense of control and self-efficacy [63], and it has been shown to exhibit a positive correlation with academic engagement (see the reviews in Derakhshan and Yin [64]). For instance, Mazer [65] evaluated the ability of pride to predict engagement among undergraduate students. The results implied that pride could play a significant role in improving undergraduate students’ engagement. Derakhshan and Yin [64] investigated the ability of positive emotions to predict engagement among language learners. These authors revealed that students who feel a sense of pride in the classroom are more likely to participate actively in the learning process. Luo and Luo [66] studied the relationships between achievement emotions and engagement in the context of Singaporean students, and the results showed that pride was positively associated with cognitive engagement. According to Alpaslan and Ulubey [67], pride increases students’ cognitive engagement.

Anxiety is a deconstructive emotion that is frequently experienced by students and can cause them to disagree [68]. This conclusion has been confirmed by many studies. For example, Shirvan and Taherian [69] suggested that a decrease in students’ anxiety leads to increased engagement. Students’ anxiety is also a strong inverse predictor of their engagement [65]. Bhuttah et al. [70] provided further evidence to support this claim by highlighting the significant negative effect of anxiety on student engagement in the context of science education. Kang et al. [71] demonstrated that anxiety has a negative relationship with academic engagement, thereby emphasizing the detrimental impact of anxiety on student engagement. Moreover, Hu et al. [72] observed a negative correlation between anxiety and academic engagement, thus indicating that anxiety can hinder students’ engagement in academic activities.

Overall, these prior studies have suggested that pride should increase cognitive engagement anxiety and decrease cognitive engagement. Thus, the following hypotheses are proposed for this study:

**H3.**  *Pride (PRI) positively influences cognitive engagement.*

**H4.**  *Anxiety (ANX) negatively impacts cognitive engagement.*

### 2.4. Perceptions of the Smart Classroom Environment and Achievement Emotions

A student’s emotions are influenced by various environmental factors [73], and such environmental factors have been identified as proximal influences of emotions [74]. Research has indicated that the classroom environment plays a crucial role in shaping students’ learning experiences, emotions, comfort levels, and concentration [47]. Frenzel, Pekrun, and Goetz [73] conducted a comprehensive study and used a multilevel approach to investigate the relationship between classroom environments and emotions in the context of mathematics; the results highlighted strong connections between environmental factors and emotional responses. Specifically, factors such as perceived teaching quality and peers’ attitudes toward mathematics were shown to be positively associated with feelings of enjoyment but negatively associated with emotions such as anxiety, anger, and boredom. Avar’s research on high school students in biology classes revealed that a conducive learning atmosphere increases such students’ positive emotions and decreases their negative emotions [75]. Similarly, Kohoulat et al. [76] investigated medical students, revealing that students’ perceptions of the learning environment have significant positive predictive effects on students’ feelings of pride and significant negative predictive effects on their feelings of anxiety.

Moreover, Goetz et al. [77] identified a positive correlation between students’ perceptions of learning environments and their individual feelings of pride, while Kong and Zeng [78] reported a positive relationship between perceived environmental uncertainty and anxiety levels among university students. In terms of technology-enhanced learning environments, the potential benefits of a flipped learning environment may cause learners to experience a greater sense of control and more positive value appraisals than in traditional classroom settings, thereby potentially reducing negative emotions such as anxiety [79].

These inconsistent empirical findings have highlighted the positive relationship between pride and learning environment preferences and the negative relationship between anxiety and perceptions of the learning environment. Based on these insights, the following hypotheses are proposed:

**H5.**  *Perceptions of a smart classroom environment positively impact feelings of pride.*

**H6.**  *Perceptions of a smart classroom environment negatively affect feelings of anxiety.*

### 2.5. The Perceived Usefulness of Mobile Technology and Achievement Emotions

Researchers have suggested that if students perceive technology as useful for their learning, they may experience more positive achievement emotions, such as pride or enjoyment, when they successfully use technology to complete academic tasks [80,81]. Butz et al. [82] investigated the role of emotions in technology-mediated synchronous hybrid learning environments and revealed that online students reported higher levels of technology-related anxiety than did students in traditional educational settings. Research on virtual classrooms has shown that students’ perceptions of the usefulness of technology can mitigate the negative impact of social disconnectedness on their achievement emotions [83]. While the use of the internet and technology can help alleviate anxiety during the learning process [84], the inappropriate use of mobile technologies may lead students to feel purposeless, anxious, and disengaged [49]. A study focusing on the relationship between digital technology use and students’ achievement emotions in the contexts of science and mathematics highlighted a positive correlation between digital technology use and students’ feelings of enjoyment and pride. Notably, while no direct negative association was observed between students’ digital technology use and feelings of anxiety and boredom, digital technology use did have an indirect effect on students’ cognitive appraisals [85].

Based on the literature reviewed above, we propose the following hypotheses:

**H7.**  *The perceived usefulness of mobile technology positively influences feelings of pride.*

**H8.**  *The perceived usefulness of mobile technology negatively affects feelings of anxiety.*

### 2.6. The Mediating Role of Achievement Emotions

Achievement emotions are triggered by various types of appraisals of the environment and influence students’ engagement [57]. Kong and Zeng [78] reported a positive relationship between perceived environmental uncertainty and anxiety among university students, which subsequently had a negative impact on academic engagement. This finding suggests that external factors, such as environmental uncertainty, can exacerbate students’ levels of anxiety, thereby ultimately having a negative effect on student engagement. When students perceive mobile technology as useful, this situation can lead to positive activating emotions, which have been shown to preserve individuals’ cognitive resources, direct their attention, and promote their motivation and deep learning. Moreover, the perceived usefulness of mobile technology can also mitigate negative activating emotions such as anxiety, which are typically associated with reduced cognitive resources, decreased motivation, and surface learning [80,86].

Based on the hypotheses proposed above and the results of a review of the relevant literature, we believe that achievement emotions mediate the relationships among students’ perceptions of smart classroom environments, perceived usefulness of mobile technology, and cognitive engagement. Thus, we propose the following hypotheses:

**H9.**  *Pride mediates the relationship between students’ perceptions of smart classroom environments and cognitive engagement.*

**H10.**  *Pride mediates the relationship between students’ perceived usefulness of mobile technology and cognitive engagement.*

**H11.**  *Anxiety mediates the relationship between students’ perceptions of smart classroom environments and cognitive engagement.*

**H12.**  *Anxiety mediates the relationship between students’ perceived usefulness of mobile technology and cognitive engagement.*

**Figure 1:**
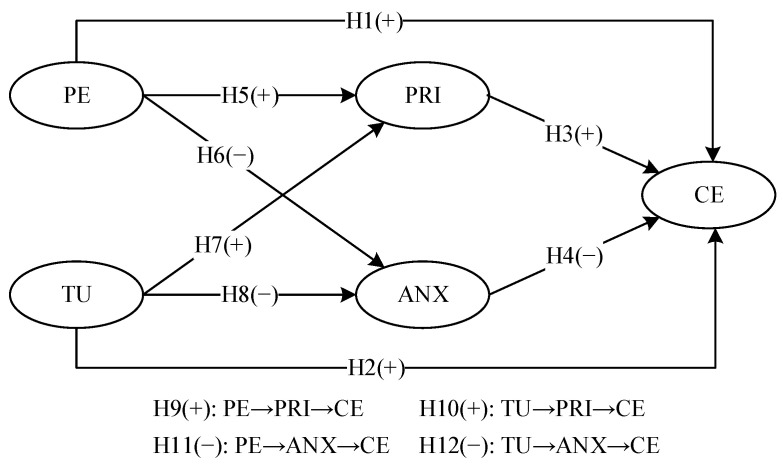
The hypothesized model.

The complete hypothesized model pertaining to these variables is presented in Figure 1.

## 3. Methodology

Structural equation modeling (SEM) analysis was employed as the primary research methodology in this study. Self-reported data regarding students’ perceptions of the smart classroom environment, perceived usefulness of mobile technology, pride, anxiety, and cognitive engagement were collected from college students using an online questionnaire. Following the data collection process, the researchers utilized SEM analysis to explore the relationships among the variables of interest, including both direct and indirect effects. Based on this analysis, the researchers evaluated the hypothesized relationships and assessed the overall fit of the proposed model.

### 3.1. Research Context

Since this study did not employ a specific sampling frame, it was not feasible to implement a random sampling approach that could take into account all potential learners in smart classroom settings located in mainland China. Therefore, the survey participants were college students from the authors’ schools who had previous learning experience in smart classrooms. The questionnaire was distributed through an online survey website and was sent to students through a WeChat community, which was built into the class unit. All participants in this study were informed that their participation was optional, and they retained the right to withdraw from the study at any stage without facing any consequences. Furthermore, participants were ensured that any personally identifiable details would remain anonymous in all associated publications and presentations.

### 3.2. Participants

For the purpose of this study, a sample consisting of 1293 college students from China was selected, and a valid sample of 1076 participants was retained, for an effective sample recovery rate of 83.22%. Among participants, 212 were male (19.7%), while 864 were female (80.3%). In terms of grade distribution, the majority of participants were in Grade 2 (42.5%) or Grade 3 (44.2%), followed by Grade 1 (12.5%) and Grade 4 (0.8%). According to the educational system in China, high school students typically enter university at the age of 18, and our investigation was conducted during the second semester of the university’s freshmen enrollment. Therefore, in this study, the age distribution of the student samples generally follows that freshmen are 19 years old, sophomores are 20, juniors are 21, and seniors are 22.

### 3.3. Instrument

The instrument used in this research study was a 25-item questionnaire (see Appendix A). The questionnaire consisted of two sections. The first section focused on demographic questions, including questions about participants’ birth sex and grade; the aim of this section was to collect students’ background information. In addition to the demographic information of the respondents, the second section included items scored on a five-point Likert scale, which were used to assess participants’ perceptions of the smart classroom environment, perceived usefulness of mobile technology, pride, anxiety, and cognitive engagement. All construct measures were taken from existing instruments that have exhibited good validity and reliability in previous studies. We made minor modifications to the instrument items to adopt them to the current research context. In particular, the items used to measure students’ perception of smart classroom environments were adapted from the questionnaire compiled by Dai et al. [87]; those used to measure the perceived usefulness of mobile technology were adapted from the literature on the technology acceptance model (TAM) [88,89]; the two parts of the achievement emotion questionnaire developed by Bieleke et al. [90] pertaining to “pride” and “anxiety” were used to measure the emotions that students may experience in the smart classroom; and the items used to measure student cognitive engagement were adapted from an instrument that was validated by Sun and Rueda [91].

### 3.4. Data Analysis Method

The methodology used for the data analysis consisted of a confirmatory factor analysis to validate the constructs and an evaluation of internal consistency by reference to Cronbach’s α and reliability indices such as composite reliability (CR) and average variance extracted (AVE). Descriptive, correlation, and variance analyses were performed using SPSS 23.0 software. Subsequently, the proposed structural model was tested using AMOS 28.0 software.

## 4. Results

### 4.1. Reliability and Validity Analysis

The reliability of the study instrument was measured by reference to Cronbach’s α coefficient, and the structural validity of the questionnaire was determined by reference to convergent and discriminant validity. The detailed reliability and validity values for the questionnaire are presented in Table 1. The reliability test for the Cronbach’s α coefficients for all the constructs ranged between 0.939 and 0.960, all of which were above 0.7 [92], thus indicating that these constructs exhibited a high degree of reliability.

The structural validity of the questionnaire was assessed based on its convergent and discriminant validity [93]. Convergent validity evaluates the extent of shared variance among the indicators associated with the underlying construct and can be determined by analyzing the standardized factor loadings, composite reliability (CR) values, and average variance extracted (AVE) values of the items. Bagozzi and Yi [94] suggested that acceptable convergent validity is indicated by factor loadings above 0.6, a CR value above 0.7, and an AVE value higher than 0.5. In this study, the dimensions included in the questionnaire met these criteria, and the questionnaire thus exhibited satisfactory convergent validity.

Discriminant validity refers to the degree to which measures of different constructs are not correlated with one another. Fornell and Larcker [95] proposed that discriminant validity can be verified when the square root of the AVE value for a particular construct is greater than its correlation with other constructs. In our study, the square root of the AVE values for all the constructs included in the questionnaire fell within the range of 0.739 to 0.916, thereby exceeding the corresponding correlation coefficients. The results indicated that the questionnaire exhibited good discriminant validity.

### 4.2. Descriptive Statistics, Correlation Analysis, and Individual Differences

The overall descriptive statistics and correlations pertaining to students’ perceptions of the smart classroom environment, perceived usefulness of mobile technology, pride, anxiety, and cognitive engagement in the learning process are presented in Table 2. These data demonstrate that students rated their overall feelings regarding the smart classroom environment (M = 4.34), perceived usefulness of technology (M = 4.31), pride experienced in the smart classroom (M = 4.24), and cognitive engagement in learning in the smart classroom environment (M = 4.26) as better than the standardized values, while their perceived anxiety in the smart classroom (M = 2.11) was low. The positive correlations observed between pride and perceptions of the smart classroom environment, the perceived usefulness of mobile technology, and cognitive engagement (which ranged from 0.528 to 0.746), alongside the negative correlations observed between anxiety and the other variables (ranging from −0.274 to −0.174), are consistent with the theoretically expected valence.

Furthermore, we evaluated the differences in all variables across sex and grade levels among the students. Given the use of a Likert scale and the non-normal distribution of the sample data in our study, we opted for nonparametric tests for independent samples. Specifically, we applied the Mann–Whitney U test to ascertain the differences between the two independent groups by gender, and the Kruskal–Wallis test to explore the differences among four independent grade-level samples. H value is a test statistic used in the Kruskal-Wallis test, If the calculated H value is large, it indicates that at least one group is different from the others, *p*-value indicates that whether the differences between the groups are statistically significant. The descriptive statistics and differences between boys and girls pertaining to all measures are presented in Table 3. The results showed that there was no significant gender difference in these variables. Descriptive statistics and differences among different grade levels for all measures are presented in Table 4. The results of multiple independent samples tests show that all variables exhibited significant differences at the grade level, thus indicating that the students’ grade level had significant effects on the smart classroom learning experience and effectiveness.

### 4.3. Structural Equation Model Analyses and Hypothesis Testing

Before proceeding with the structural equation model analysis, an assessment of potential multicollinearity among the independent variables was conducted. Based on established criteria, a tolerance value below 0.1 or a variance inflation factor (VIF) higher than 10 was taken to indicate the presence of covariance among the independent variables. The results of our data analysis revealed that all independent variables exhibited tolerances greater than 0.1 (PE = 0.529, TU = 0.323, PRI = 0.443, ANX = 0.928) and VIFs below 10 (PE = 1.889, TU = 3.098, PRI = 2.259, ANX = 1.077). Consequently, our findings indicated the absence of multicollinearity among the independent variables, thus allowing us to proceed with the path analysis based on structural equation modeling.

Path analysis was first conducted for the hypothetical model proposed in this study with the goal of assessing the mediating effects of positive emotional pride and negative emotional anxiety on students’ perceptions of the smart classroom environment, perceived usefulness of mobile technology, and cognitive engagement. Using path analysis and maximum likelihood, the fit of the model was assessed by reference to χ^2^/df, the comparative fit index (CFI), the goodness-of-fit index (GFI), the adjusted goodness-of-fit index (AGFI), the Tucker–Lewis index (TLI), and the root mean square error of approximation (RMSEA). The goodness of fit exhibited by the hypothesized model in this study was χ^2^/df = 3.282, CFI = 0.983, GFI = 0.947, AGFI = 0.933, TLI = 0.981, and RMSEA = 0.046, thus indicating that the fit was acceptable.

**Figure 2:**
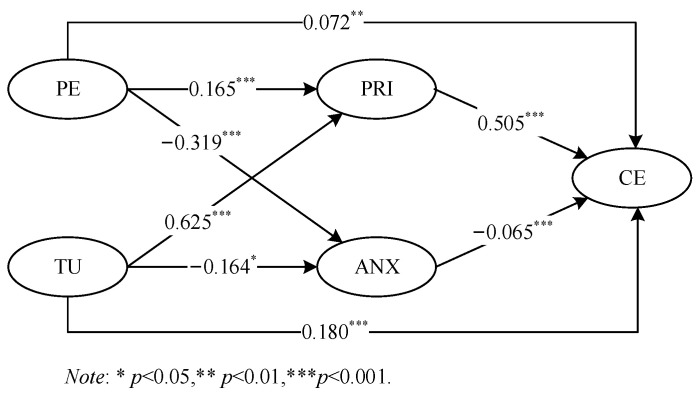
The path coefficients of the model.

The direct path coefficients of the initial structural model are presented in Figure 2 and Table 5. First, the results indicated that perceptions of the smart classroom environment (β = 0.072, *p* = 0.006), perceived usefulness of mobile technology (β = 0.180, *p* < 0.001), and pride (β = 0.505, *p* < 0.001) positively predict cognitive engagement, while anxiety (β = −0.065, *p* < 0.001) negatively predicts cognitive engagement. In addition, perceptions of the smart classroom environment (β = 0.165, *p* < 0.001) and the perceived usefulness of mobile technology (β = 0.625, *p* < 0.001) were positively related to pride but negatively associated with anxiety (PE: β = −0.319, *p* < 0.001; TU: β = −0.164, *p* = 0.018).

Second, based on the bias-corrected bootstrapping test results (see Table 6), the indirect effects of perceptions of the smart classroom environment on cognitive engagement via achievement emotions (PRI: indirect effect = 0.083, 95% CI = [0.041, 0.135]; ANX: indirect effect = 0.021, 95% CI = [0.010, 0.038]) were significant. Similarly, the indirect effects of the perceived usefulness of mobile technology on cognitive engagement via achievement emotions (PRI: indirect effect = 0.315, 95% CI = [0.237, 0.396]; ANX: indirect effect = 0.011, 95% CI = [0.000, 0.024]) were significant. Overall, these results revealed that pride and anxiety mediate the relationships among perceptions of the smart classroom environment, the perceived usefulness of mobile technology, and learning engagement.

Finally, the hypothesis testing results presented in Table 5 and Table 6 demonstrate that all twelve hypotheses proposed in the present study were supported. In addition, the standardized path coefficient findings, which are presented in Table 5, reveal that among the four variables influencing student cognitive engagement, positive emotions (pride) are the most significant, followed by the perceived usefulness of mobile technology. Notably, the indirect effect of pride was also the strongest.

## 5. Discussion

Engaging students has been widely recognized as crucial with regard to attempts to promote student learning in diverse environmental settings [7]. In the present study, students reported a high level of cognitive participation in smart classroom environments, thus indicating that they can participate more effectively in the learning process in the context of the smart classroom. The results of previous research have suggested a relationship between student engagement and sex, in which context boys typically exhibit lower levels of engagement [96,97,98]. Unexpectedly, no sex differences in students’ cognitive engagement in smart classrooms were observed in our study, a conclusion that differs from the findings of previous research. In terms of grade level, our analyses confirmed the results of previous studies [98,99] by indicating that students’ cognitive engagement in smart classrooms varies at different grade levels and exhibits a decreasing trend.

The present study examined the relationships among college students’ perceptions of the smart learning environment, perceived usefulness of mobile technology, achievement emotions, and their cognitive engagement in smart classrooms. The results revealed that students’ experiences with the learning environment in smart classrooms and the perceived usefulness of mobile technology as well as the resulting positive and negative emotions have significant predictive effects on their cognitive engagement, and achievement emotions mediate the relationships among perceptions of the learning environment, the usefulness of mobile technology, and cognitive engagement. In addition, Pekrun et al. [100] noted that negative emotions such as anxiety and anger can energize students, thus increasing rather than decreasing their engagement. Our findings showed that pride, as a positive emotion, affects student cognitive engagement, whereas anxiety, as a negative emotion, is negatively associated with cognitive engagement; furthermore, the impact of positive emotion is greater than that of negative emotion. Our results largely converged with the extant literature and theoretical evidence available on this topic.

Our research findings provide evidence to support the claim that technology-based environments positively influence students’ cognitive engagement [18,49]. Due to its collaborative and communicative features, mobile technology can potentially enhance these emotions by providing students with a sense of control and value during the learning process. When students perceive mobile technology as useful, this attitude can elicit positive activating emotions such as enjoyment and hope, which are known to preserve individuals’ cognitive resources, direct their attention, and promote their motivation and deep learning. Moreover, the perceived usefulness of mobile technology can mitigate negative activating emotions such as anxiety, which are typically associated with reduced cognitive resources, decreased motivation, and surface learning [101]. By integrating mobile technology effectively, educators can potentially establish a more positive learning environment that encourages adaptive achievement emotions, thereby promoting learning and achievement.

Kirkwood [102] claimed that technology use does not guarantee active student engagement. Instead, meticulous planning, effective pedagogy, and suitable tools are crucial, as mentioned by Englund et al. [103]. Without meticulous planning and effective pedagogy, technology can result in disengagement and act as a hindrance to rather than a facilitator of learning [104]. Therefore, in a smart classroom, which represents a technology-supported learning environment, the learning process should be combined with instruction methods, reasonable planning, and the use of technology, thus allowing students to feel that the use of technology is useful with regard to promoting the generation of positive emotions, reducing negative emotions, and improving their engagement.

## 6. Conclusions

The paper explores the interrelationship among smart classroom environments, mobile technology, achievement emotions, and students’ cognitive engagement. The findings showed that students report high levels of cognitive engagement in these settings. It challenges previous research by not finding sex differences in cognitive engagement levels. The study confirms that cognitive engagement varies by grade and decreases with higher grade levels. The research also reveals that students’ perceptions of the smart learning environment, the perceived usefulness of mobile technology, and their emotions significantly predict their cognitive engagement, with achievement emotions acting as a mediator. Positive emotions like pride enhance cognitive engagement, while negative emotions like anxiety reduce it, with the positive impact outweighing the negative. This paper supports the idea that technology usage can improve cognitive engagement but emphasizes that effective integration with pedagogy and planning is necessary to avoid disengagement and promote a positive learning environment that fosters adaptive emotions and deep learning.

## 7. Implications

A technology-enhanced environment elicits positive emotions from learners and alleviates their negative emotions by providing them with personalized and adaptive learning experiences as well as by promoting social interactions. Thus, smart classroom environments play a crucial role in influencing students’ achievement emotions by promoting positive emotions and minimizing negative emotions. These learning environments help cultivate an atmosphere that benefits students’ engagement, ultimately impacting their learning experiences and academic success. For example, students’ anxiety can be reduced by allowing them to use their own mobile devices and ensuring that their technology use is helpful with regard to their learning, thus establishing a more supportive learning environment. Hence, a well-designed smart classroom should provide a comfortable, safe, and stimulating environment that encourages active learning and collaboration.

### 7.1. Implications for Researchers

Researchers should focus on the design and impact of technology-enhanced environments on the achievement emotions and engagement of students. The study of personalized and adaptive learning experiences, as well as the role of social interactions based on mobile technology in smart classrooms, is crucial. Understanding how these environments can promote positive emotions and mitigate negative ones, such as anxiety, is essential for developing effective educational strategies. Additionally, the exploration of the integration of interactive technologies in smart classrooms to enhance student engagement is a significant area for further investigation.

### 7.2. Implications for Practice

One key mechanism that can enhance student engagement in smart classrooms is the use of interactive technologies. These technologies, such as interactive whiteboards, touchscreens, and learning management systems, can provide students with a more dynamic and engaging learning experience. By allowing students to participate actively in the learning process, interactive technologies can foster a sense of agency and ownership over their learning among students, which can lead to higher levels of engagement and motivation. Given that the improper use of mobile devices can become a distraction in the learning environment [49], instructors in a smart classroom environment must ensure that students perceive the usefulness of mobile devices by designing technology-based learning activities (e.g., activities including the use of mobile response systems [105]; mobile technology-based interactive activities such as polls, exercises, quizzes and games [106]; and online interactions that take place via mobile applications [107]), with the goals of enhancing students’ cognitive engagement and generating positive emotions that can affect their cognitive engagement indirectly. This factor is crucial for teachers who seek to use mobile devices and achieve greater success in smart classroom environments.

### 7.3. Implications for Decision-Makers

By integrating mobile technology effectively, educators can potentially establish a more positive learning environment that encourages positive achievement emotions, thereby promoting learning and achievement. Administrators and policymakers in education must recognize the importance of integrating mobile technology effectively into the learning environment. They should invest in activities that are designed to enhance cognitive engagement and generate positive emotions, such as mobile response systems, interactive activities, and online interactions via mobile applications.

## 8. Limitations

Our study has several limitations that should be addressed in future research. First, the sample was obtained using online data collection procedures. Although every effort was made to provide detailed administrative protocols, it was impossible to ensure complete fidelity with the desired survey procedures. Second, our research relied heavily on self-reported data, thus potentially introducing measurement errors due to reporter bias or discrepancies in the individuals’ reports concerning their self-construal. To address this issue, the incorporation of multiple measures is imperative to a comprehensive assessment. For example, explorations of student engagement in a smart learning environment could benefit from multimodal data, which can be collected using advanced artificial intelligence technologies. This approach could involve the use of various tools, such as the Internet of Things, perception technology, video recording technology, image recognition technology, and platform acquisition technology. By taking advantage of multisource, heterogeneous, and multimodal big data (e.g., capturing gestures, facial expressions, body language, and verbal interactions) concerning students’ learning processes, a more nuanced understanding of students’ engagement levels can be obtained. Third, our research explored only the partial learning environment experiences of students and did not consider individual factors, such as students’ prior knowledge or experiences with technology and technology-enriched learning environments or the contexts of specific subjects (e.g., English as foreign language learning, mathematics). However, the control-value theory framework posits that achievement emotions are shaped by environmental, situational, and individual factors that are experienced at school [63]. Importantly, the effectiveness of mobile technology with regard to enhancing achievement emotions can be influenced by various factors, including the educational context, the specific pedagogical use of the technology in question, and individual differences among students. Further research is needed to improve our understanding of the nuances that characterize this relationship and to develop targeted strategies that can take advantage of the potential of mobile technology to support students’ emotions and achievement.

## Figures and Tables

**Table 1:** Questionnaire constructs, reliability, and validity.

Constructs	Items	Cronbach’s α	Factor Loading	CR	AVE	$\sqrt{\mathrm{AVE}}$
PE	5	0.943	0.737–0.927	0.947	0.783	0.885
TU	4	0.952	0.789–0.886	0.908	0.712	0.844
PRI	4	0.956	0.710–0.759	0.828	0.547	0.739
ANX	4	0.939	0.862–0.943	0.954	0.840	0.916
CE	5	0.960	0.873–0.901	0.947	0.782	0.885

**Table 2:** Means, standard deviations, and correlations among variables.

Variable	Statistic		Correlations			
	Mean	SD	1	2	3	4
1. PE	4.34	0.75	-			
2. TU	4.31	0.70	0.644 **	-		
3. PRI	4.24	0.74	0.528 **	0.662 **	-	
4. ANX	2.11	1.12	−0.251 **	−0.221 **	−0.174 **	-
5. CE	4.26	0.70	0.535 **	0.644 **	0.746 **	−0.274 **

Note: ** *p* < 0.01.

**Table 3:** Means, standard deviations, and differences between boys and girls for all measures.

Construct	Sex				*p*
	Boys (*n* = 212)		Girls (*n* = 864)		
	Mean	SD	Mean	SD	
PE	4.309	0.901	4.346	0.709	0.425
TU	4.333	0.816	4.304	0.672	0.099
PRI	4.259	0.855	4.231	0.714	0.078
ANX	2.337	1.323	2.054	1.051	0.051
CE	4.261	0.820	4.254	0.667	0.316

**Table 4:** Means, standard deviations, and differences between grades for all measures.

Construct	Grade Level								H
	Grade 1 (*n* = 135)		Grade 2 (*n* = 457)		Grade 3 (*n* = 476)		Grade 4 (*n* = 8)		
	Mean	SD	Mean	SD	Mean	SD	Mean	SD	
PE	4.391	0.707	4.162	0.789	4.494	0.689	4.375	0.627	53.931 ***
TU	4.302	0.812	4.192	0.703	4.423	0.651	4.406	0.626	28.848 ***
PRI	4.320	0.814	4.124	0.734	4.319	0.722	4.344	0.654	24.485 ***
ANX	1.693	0.850	2.345	1.114	2.000	1.132	2.219	1.250	52.867 ***
CE	4.397	0.640	4.136	0.696	4.335	0.698	3.975	0.922	28.983 ***

Note: *** *p* < 0.001.

**Table 5:** The path coefficients of the initial structural model and hypothesis testing results.

Hypotheses	Paths	Unstandardized Path Coefficients	Std. Error	*p* Value	95% CI		Std. Beta	Result
					Lower	Upper		
H1(+)	PE→CE	0.072	0.026	0.006 **	0.013	0.136	0.079	Supported
H2(+)	TU→CE	0.180	0.031	***	0.083	0.276	0.193	Supported
H3(+)	PRI→CE	0.505	0.027	***	0.415	0.609	0.578	Supported
H4(−)	ANX→CE	−0.065	0.012	***	−0.094	−0.041	−0.112	Supported
H5(+)	PE→PRI	0.165	0.035	***	0.084	0.272	0.157	Supported
H6(−)	PE→ANX	−0.319	0.069	***	−0.504	−0.159	−0.200	Supported
H7(+)	TU→PRI	0.625	0.037	***	0.520	0.729	0.587	Supported
H8(+)	TU→ANX	−0.164	0.069	0.018 *	−0.318	−0.003	−0.102	Supported

Note: * *p* < 0.05, ** *p* < 0.01, *** *p* < 0.001.

**Table 6:** A bias-corrected bootstrap test of the mediating effects of achievement emotions and hypothesis testing results.

Hypotheses	Paths	Effects	95% CI		Results
			Lower	Upper	
H9(+)	PE→PRI→CE	0.083 ***	0.041	0.135	Supported
H10(−)	PE→ANX→CE	0.021 ***	0.010	0.038	Supported
H11(+)	TU→PRI→CE	0.315 ***	0.237	0.396	Supported
H12(−)	TU→ANX→CE	0.011 *	0.000	0.024	Supported

Note: * *p* < 0.05, *** *p* < 0.001.

## Data Availability

The datasets used and analyzed during the current study are available from the corresponding authors upon reasonable request.

## Appendix A

**Table A1:** Items pertaining to environmental perception, technical usefulness, pride, anxiety, and cognitive engagement.

Dimension	No.	Item
Environmental perception (PE)	1	The infrastructure in smart classrooms (such as desks and chairs, lighting, sound, air conditioning, curtains, displays, cameras, network equipment, and central control consoles) is well configured.
	2	The smart classroom is equipped with a complete set of intelligent terminal equipment (including tablet computers, PC terminals, and electronic brands), multimedia teaching equipment (including teaching platform controllers, liquid crystal touch screens, recording and broadcasting equipment, and wireless microphones), and Internet of Things equipment (including equipment pertaining to the temperature, humidity, and light sensors).
	3	In the smart classroom, students’ intelligent login, intelligent grouping, and intelligent acquisition of information and resources can be realized.
	4	In the smart classroom, it is very convenient to interact with teachers through their own mobile phones or tablets.
	5	I prefer teachers to employ various forms of classroom teaching in the smart classroom and to interact with us via the online learning platform after class.
Technical usefulness (TU)	6	Using mobile phones or computers to complete learning tasks is very helpful with respect to my learning.
	7	Using mobile phones or computers to complete learning tasks can help me learn more effectively.
	8	Using mobile phones or computers to complete learning tasks can improve learning efficiency.
	9	Using mobile phones or computers to complete learning tasks allows me to exhibit better learning performance.
Pride (PRI)	10	In the smart classroom, I am proud of myself.
	11	I am proud of what I have mastered in the smart classroom.
	12	The achievements I have made in the smart classroom have inspired me to continue to study diligently.
	13	I will be very proud when I perform well in the smart class.
Anxiety (ANX)	14	In the smart classroom, I feel anxious.
	15	Before participating in the wisdom class, I worry about whether I understand the learning materials.
	16	Sometimes I worry because of tension and would rather skip class.
	17	I will be very psychologically nervous in the smart classroom.
Cognitive engagement (CE)	18	I will try to use the internet, television, books, magazines, and other media to find and learn knowledge related to the course.
	19	When I am reading the course materials, I ensure that I understand the knowledge by engaging in self-questioning.
	20	I will consult more information to help me understand the course knowledge.
	21	When I encounter knowledge in class, I will think of various ways of clarifying it after class.
	22	I will discuss what I have learned in class with my classmates or teachers.
